# The Role of Specialized Instruments for Advanced Endoscopic Resections in Gastrointestinal Disease

**DOI:** 10.3390/life13112177

**Published:** 2023-11-07

**Authors:** Markus Brand, Karl-Hermann Fuchs, Joel Troya, Alexander Hann, Alexander Meining

**Affiliations:** Interventional and Experimental Endoscopy (InExEn), Department of Internal Medicine II, Gastroenterology, University of Würzburg, 97070 Würzburg, Germany; brand_m@ukw.de (M.B.); troyasebas_j@ukw.de (J.T.); meining_a@ukw.de (A.M.)

**Keywords:** interventional endoscopy, therapeutic endoscopy, endoscopic graspers, ESD, GI endoscopy

## Abstract

Introduction: Advanced endoscopic therapy techniques have been developed and have created alternative treatment options to surgical therapy for several gastrointestinal diseases. This work will focus on new endoscopic tools for special indications of advanced endoscopic resections (ER), especially endoscopic submucosal dissection (ESD), which were developed in our institution. This paper aims to analyze these specialized instruments and identify their status. Methods: Initially, the technical process of ESD was analyzed, and the following limitations of the different endoscopic steps and the necessary manipulations were determined: the problem of traction–countertraction, the grasping force needed to pull on tissue, the instrument tip maneuverability, the limited angulation/triangulation, and the mobility of the scope and instruments. Five instruments developed by our team were used: the Endo-dissector, additional working channel system, external independent next-to-the-scope grasper, 3D overtube working station, and over-the-scope grasper. The instruments were used and applied according to their special functions in dry lab, experimental in vivo, and clinical conditions by the members of our team. Results: The Endo-dissector has a two-fold function: (1) grasping submucosal tissue with enough precision and strength to pull it off the surrounding mucosa and muscle, avoiding damage during energy application and (2) effectively dividing tissue using monopolar energy. The AWC system quickly fulfills the lack of a second working channel as needed to complete the endoscopic task on demand. The EINTS grasper can deliver a serious grasping force, which may be necessary for a traction–countertraction situation during endoscopic resection for lifting a larger specimen. The 3D overtube multifunctional platform provides surgical-like work with bimanual-operated instruments at the tip of the scope, which allows for a coordinated approach during lesion treatment. The OTSG is a grasping tool with very special features for cleaning cavities with debris. Conclusions: The research and development of instruments with special features can solve unmet needs in advanced endoscopic procedures. The latter may help to increase indications for the endoscopic resections of gut lesions in the future.

## 1. Introduction

Advanced endoscopic therapy techniques have been developed and have created attractive and alternative treatment options to surgical therapy for several gastrointestinal diseases, such as tumor resections, myotomies, and bypassing malignant stenoses [1,2,3,4,5,6,7,8]. The more these endoscopic procedures are specialized, the more valuable specialized endoscopic instruments become for certain, possibly infrequent, situations. Their use may be at least helpful, if not quite important, to facilitate the necessary maneuvers [9,10].

While Research and Development units run by large international companies in the endoscopy market are more interested in the development of instruments for future mass production and may not bother with small instrument series, research institutes associated with university departments may focus on the development of specialized small-series instruments for very dedicated purposes [10,11]. High-volume endoscopic therapists may have an unmet need for such instruments, as they may notice certain shortcomings during the many procedures, experiencing a lack of speed or manipulation abilities using traditional instruments. Several new ideas and technologies regarding endoscopic tools are emerging to improve the therapeutic efficacy of these procedures [12,13,14]. 

The basic step towards detecting these needs is an analysis of the different procedural steps of endoscopic procedures, obtaining the flaws and obstacles that are found during these interventional therapies. Some of the most frequently performed interventional endoscopic procedures are endoscopic resections (ER) using different techniques such as fluid-sustained endoscopic mucosal resection (EMR), endoscopic submucosal dissections (ESD), and endoscopic full-thickness resections (EFTR) [1,2,3,4,5,6,7,8]. ESD and EFTR have emerged as valuable therapeutic options, which can be used in many clinical cases, replacing more invasive procedures such as partial or complete organ removals via gastrointestinal surgery [1,2,3,4,5,6,7,8]. This progress in advanced therapeutic endoscopy was made possible with the development of new technology, creating new concepts and new instruments for flexible endoscopic use, providing special functions to enable complex maneuvers, and/or applying necessary technical features. Since the concept and design of a modern endoscope do not differ much from the endoscopes 30 years ago, the main advances in developing techniques are based on innovative instruments and emerging concepts of procedural techniques.

This work was focused on a few of these new endoscopic tools for special indications, which were developed in our institution [15,16,17,18,19]. This paper aims to review and analyze the status of these five specialized instruments in the process of advanced endoscopic therapies, especially ESD, based on an identification of some unmet needs and using specific instruments to compensate for the shortcomings of traditional techniques.

## 2. Methods

Initially, the technical processes of ESDs and the removal of specimens were analyzed, and the limitations of the different endoscopic steps and the necessary manipulations were documented. Many critical issues were identified, and possible improvements of the involved instruments were discussed. We must emphasize that we focused on our single-center experience, using only the instruments developed by our team.

Critical issues during advanced endoscopic resections include the problem of traction–countertraction, the necessary grasping force to pull on tissue, the instrument tip maneuverability, its dependence on the scope, the limited angulation and triangulation of the operating instruments, and the necessary mobility of the scope and instruments within the gastrointestinal (GI−) tract in oral and aboral advancements. In addition, in response to different specialized tasks, various grasping abilities, such as a mild force with a tiny tip or a large excavated grasping capacity for larger volumes, may be clinically needed. The available instruments are demonstrated in Table 1. 

The first instrument chosen for this study is the Endo-dissector (Karl Storz, Tuttlingen, Germany) [15]. The instrument has the design of a grasping tool with metal branches, which are connected to the energy supply, allowing for the tissue to be divided. The grasper has rather long and strong branches compared to regular endoscopic grasping devices, thus allowing for a strong pulling force on the tissue, which should be clearly identified and divided.

**Figure 1:**
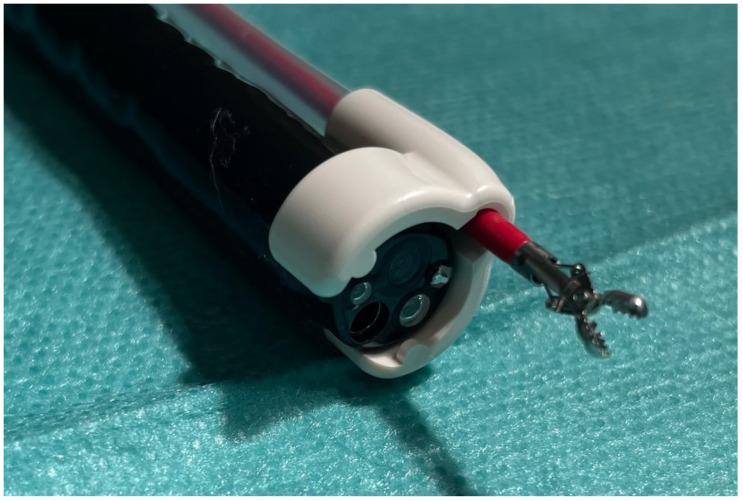
The AWC system with an additional working channel, attached to the scope, which allows for additional traction and the assistance of a second instrument.

The second instrument analyzed is the additional working channel (AWC) system, an external working channel that is closely attached to the regular scope (Ovesco Endoscopy, Tübingen, Germany) [16]. The system fits on scopes with a diameter of 8.5–13.5 mm. The system is mounted on the tip of the scope with an adjustable distance to the scope’s working channel and fixed with circular tape. The instrument size ranges to 2.8 mm in diameter (Figure 1). 

Thus, advanced endoscopic tasks can be performed using two different instruments at the same time in a regular endoscope, which may enable some minor form of triangulation and some form of traction and countertraction to be realized. With the second instrument outside the scope diameter, the distance between the two operating instruments is only slightly larger than in a two-channel endoscope.

**Figure 2:**
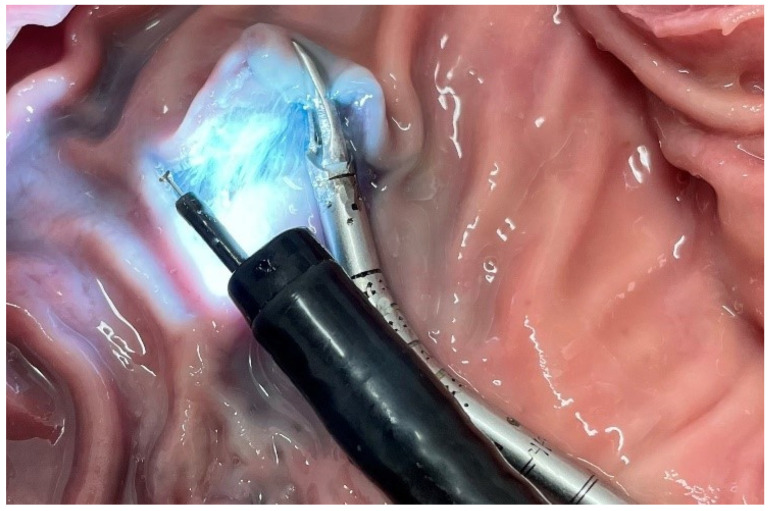
The external independent next-to-the-scope grasper (EINTS), which is operated independently of the scope and provides additional traction–countertraction.

The third instrument is the external independent next-to-the-scope (EINTS) grasping device (Fortimedix Surgical BV, Geleen, The Netherlands) [19]. Its metal shaft can be steered from outside the body via a stronghold fixed to the table (Figure 2). Since the instrument is operated entirely independently from the endoscope, all movements can be performed independently but in close coordination with the instruments through the scope, thus creating triangulation and a realistic option for traction and countertraction. In addition, the mechanical character of the system is very stable, allowing for a large grasping and pulling force.

**Figure 3:**
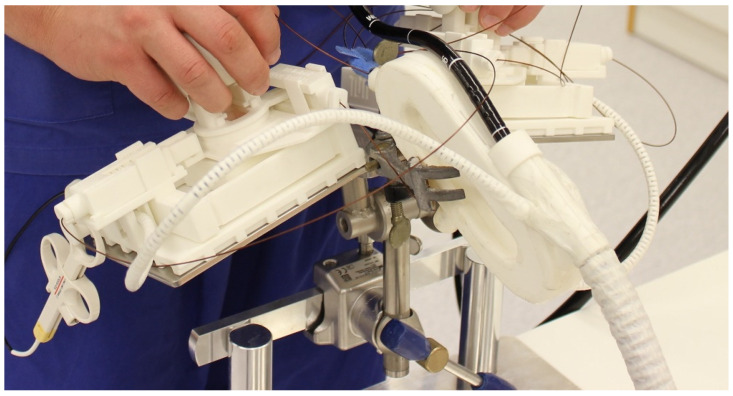
Three-dimensionally printed endoscopic manipulator system with overtube and steering mechanism of endoscopic instruments. The endoscopists operate the system via a separate working station.

The fourth instrument is the single-port overtube manipulator system (SPOT), a 3D-printed overtube system, combined with a regular endoscope for one or two instruments, developed at the Technical University in Munich, Institute of Microtechnology and Medical Device Technology and the MITI-Research Group, Munich, Germany [17]. The SPOT is a complex endoscopic working station with several elements, which are assembled to fulfill advanced endoscopic therapies (Figure 3). The overtube device is fixed on the procedure table. In the latest version, the endoscope docking and manipulator station is carried by the endoscopist around the belly, allowing for the operator to use both hands for necessary manipulations. Dissection and other manipulations using the two instruments are performed in a bimanual fashion, similar to a laparoscopic surgical paradigm. 

The fifth instrument is the over-the-scope grasper (OTSG Xcavator™) (Ovesco Endoscopy, Tübingen, Germany), designed and developed for grasping and removing larger parts of tissue, necrotic debris, and/or foreign bodies from the GI tract [18]. The system is mounted on a regular endoscope. The extra-large grasper branches are transparent to allow for a good endoscopic overview during the procedure. Its diameter is mounted, and the scope is 14.4 mm; when the grasper is opened, the diameter reaches 28.4 mm.

In our team, substantial practice with applying these instruments was noted and documented to analyze the handling, functionality, and problems, to provide more insights into the strengths and limitations of each instrument, especially regarding the different specific working areas. Our team consisted of experienced gastroenterologists, GI surgeons, engineers, and young trainees. The latter were involved, especially in the explant experiments, in instrument developments and the tests for the current analyses. We were interested in the technical shortcomings of these novices. An evaluation was performed focusing on the initially established factors that are important during advanced endoscopic resections: the problem of traction–countertraction, the necessary grasping force to pull on tissue, the instrument tip maneuverability, its dependence on the scope, the limited angulation and triangulation of the operating instruments, and the necessary mobility of the scope and instruments within the GI tract in oral and aboral advancements. These parameters were assessed and analyzed for all instruments. The results were documented semi-quantitatively to provide an overview of the different abilities and features.

The parameters for the assessment were chosen as follows: 

0 = impossible to finish the task with this instrument;

+ = completion of the task with limitations;

++ = fair performance and the completion of the task;

+++ = good to excellent performance and the completion of the task.

## 3. Results

The instruments were used and applied according to their special function in dry lab, wet lab, experimental in vivo, and clinical conditions by the members of our team over the past 2 years. The handling issues were documented, and problems and the creation of possible solutions were discussed. 

Table 2 summarizes the test experience for the current analysis with the five instruments according to the experimental and clinical application.

Table 3 summarizes the results of the analysis regarding the important issues, with a focus on the special functions of these instruments. The results were also interpreted concerning regular commercially available flexible endoscopic grasping instruments.

The Endo-dissector has a two-fold function: (1) grasping submucosal tissue with enough precision and strength to pull it off the surrounding mucosa and muscle, avoiding damage during energy application, and (2) dividing tissues effectively using monopolar energy. For this special purpose, the instrument fulfills its task excellently. It is entirely sufficient, with a good grasping force and satisfying mobility based on the scope mobility when the instrument is advanced and applied through the working channel, thus increasing the speed of the ESD because of the larger grasping area used to lift and separate a bundle of submucosal tissue with sufficient hemostasis.

The AWC system quickly fulfills the lack of a second working channel as needed to complete the endoscopic task on-demand. All available endoscopic instruments can be advanced through the channel. A dual-channel endoscope would play a similar role if available. There is even a small gain in distance between the two channels with the AWC compared to a dual-channel scope. The system increases the triangulation effect. Of course, the grasping function as such is similar to the regular instruments. Since the system mobility within the GI tract in both oral and aboral directions is hardly disturbed by the AWC, the system can be used in every section where a flexible endoscope can be applied. Thus, an ideal indication for the AWR system is the grasp-and-snare technique during endoscopic mucosal resection, which is frequently used in daily practice, or lifting the rim of a mucosal specimen during dissection of the underlying submucosal tissue.

The EINTS grasper can deliver serious grasping force, which may be necessary in a traction–countertraction situation during endoscopic resection to lift a larger specimen and/or grasp and harvest a specimen or foreign body during an advanced endoscopic therapeutic procedure. Due to its metal structure, remarkable grasping force, and ability to maneuver independently of the scope, the EINTS grasper can be applied where these features are needed. Thus, the ESD of a tumor on a larger mucosal fold may need better exposure, which can be achieved using an independent grasper reaching out from a different angle than an instrument through the working channel of the scope. This can create a true triangulation. The latter case is an ideal task and indication for the EINTS grasper.

One step further towards a multifunctional platform is the SPOT, which provides a surgical-like performance with the bimanual-operated instruments at the tip of the scope, combined with the 3D-printed overtube system. This platform allows for a coordinated approach to a lesion that needs to be endoscopically resected, since, with one arm, a grasper can be used to lift the lesion and create traction. The second arm, with an energy device, can perform the cutting and dissection at the submucosal layer or perform a full-thickness wall resection of the tumor-associated gut wall. The drawback of this system is its limited mobility within the GI tract because of the size of the overtube system and the front instrument arms. Since the system can be used with one front arm, its mobility can be improved, while, when using two front arms for the instruments, the diameter is larger. Overall, the SPOT has many good features and fulfills its tasks very effectively. However, the learning curve required to operate this system routinely requires many applications and regular training, even for specialized and highly qualified endoscopists and GI surgeons. 

The OTSG XCavator™ is a grasping tool with very special features and functions to fulfill the tasks for which it was designed. Clinical indications are the cleaning of cavities filled with debris and necrotic material after pancreatic necrosis, the endoscopic removal of large blood cloths to make the bleeding lesion visible, and the removal of foreign bodies or large specimens from the GI tract. The unique structure of the device allows for a very effective therapy for these conditions. Consequently, other features, such as triangulation or traction and countertraction, are not of interest to the OTSG and can be neglected.

The results show that several critical issues must be addressed using these different grasping systems. Specialized features may respond best to the different clinical tasks that must be solved.

## 4. Discussion

Advanced therapeutic endoscopy has emerged in the past 20–30 years as a serious player in the therapeutic spectrum of gastrointestinal disease, in both benign and malignant disorders [1,2,3,4,5,6,7,8,9,10]. The steps from endoscopic polypectomy to EMR and from ESD to EFTR are exciting and require technical advancement in instrument and device technology and, at the same time, specialization and increased workload for the endoscopist [1,2,3,4,5,6]. An ongoing adjustment and improvement of instruments is absolutely necessary. Endoscopic resections such as EMR, ESD, and EFTR are modern interventional endoscopic techniques that are increasingly used in patient care [1,2,3,4,5,6]. 

The term “Endoneering” has been introduced for a combination of endoscopic research with engineering to describe the cooperation between engineers and endoscopists [10,11,12]. Recent innovations have elevated the level of activities in endoscopic surgery or surgical endoscopy to new horizons [3,4,5,6,7]. Full-thickness resections of gut tumors, endoscopic anastomotic techniques, and the ability to suture and close the gut via endoscopic means provide the technical basis for these remarkable steps in advanced endoscopy [3,4,5,6,7,10,11,12,13,14,15,16,17,18,19]. 

These emerging technologies cause a research and development process, which requires detailed analyses of instrument actions on-site to understand the working principles, the unmet needs from the medical side, and the consulting advice of engineers to learn what is technically possible and what remains wishful thinking at present. Gastroenterology and surgical endoscopists are developing deeper insights into the technical background of instruments and will benefit from this important exposure to technical ideas when communicating with engineers. Engineers will, at the same time, benefit from this exchange of ideas and experience by learning to understand the clinical needs and technical shortcomings of these procedures and instruments [10].

The results of this study and analysis show that most instruments fulfill some basic functions depending on their design, such as all grasping instruments. However, a certain level of specialization is possible and did lead to more sophisticated features and abilities, thus providing more focus on traction–countertraction or more capacity for grasping even semisolid debris.

The increased number of publications on the traction–countertraction issue in flexible endoscopy in recent years could reflect a lack of this function in current available endoscopic instruments [20,21,22,23,24,25,26]. During high-end therapeutic endoscopies such as ESD, traction may be necessary on the gut wall or the specimen, which can be solved using the demonstrated instruments, such as the Endo-dissector and/or the EINTS. Endoscopic experts may get by without these options since they can compensate for this lack with their vast experience. For “average” endoscopists, this limitation of optimal instrument function may be critical, and the literature provides some examples of instrument optimization regarding interventional endoscopic procedures [19,20,21,22,23,24,25,26]. Several attempts were reported to create additional assisting instruments using external tools other than the regular endoscope, based on a more surgical paradigm, to facilitate endoscopic procedures [19,20,21,22,23,24,25,26]. The unmet need for a better traction–countertraction function is resolved in endoscopic practice using various grasping systems, such as the AWC system, EINTS, and SPOT [15,16,17,18,19]. These instruments can help to create enough traction, even with independent mobility from the scope, to assist in therapeutic actions during endoscopic resections [17,18,19,20]. This is especially helpful in the learning phase of these therapeutic procedures by training physicians. While AWC and EINTS clearly add a grasping tool to the regular instruments in the working channel of the scope, SPOT is a platform that allows for even more options to use different instruments during an endoscopic procedure and reminds the user of other endoscopic multitasking platforms [16,17,19].

In addition to the issue of traction–countertraction, the need for a sufficient grasping force to pull on tissue, the instrument tip maneuverability, its dependence on the scope, the limited triangulation of the operating instruments, and the necessary mobility of the scope, together with the attached instruments, can be also issues in advanced therapeutic procedures. There is a controversial discussion between some endoscopists about whether these rather sophisticated functions are really needed in flexible endoscopy, since many of these functions can be compensated by an experienced endoscopist via special angulation movements of the scope and currently available instruments via the regular working channels [2,5,9,11,12]. 

Others are searching for and testing new tools to overcome the above-mentioned limitations [10,18]. During the short hype of natural orifice transluminal endoscopic surgery, all these issues were addressed [11,12]. The necessary requirements for these procedures were defined, and the basis for the tools, instruments and the special endoscopes were discussed and published by several working groups around the world [11,12]. The answer was various endoscopic platforms with surgical abilities developed by several companies [11,27,28,29,30,31]. 

In all these platforms, multifunctional instruments based on a more or less stable basis could be manipulated from outside the body, while a flexible operating scope with several end effectors was introduced in a possible patient [11,27,28,29,30,31]. Early in clinical experience, it became obvious that the handling of these complex and sophisticated platforms and instruments required a long learning curve, even for the most dedicated and experienced endoscopists [11,27,28,29,30,31]. It became obvious that only a few of the most dedicated endoscopists, gastroenterologists, and surgeons could perform these complicated procedures. In addition, it was quite cumbersome to establish evidence that these “scar-free” procedures provided a true advantage for the involved patients [11,27,28,29,30,31,32].

Furthermore, industry and corporate partners withdrew their research and development efforts rather quickly, since these new procedures did not generally penetrate the worldwide market, as large investments were required to bring these platforms to a market level with sufficient clinical distribution [11,27,28,29,30,31].

Subsequently, endoscopists were looking for simpler solutions to the above-mentioned problems and issues, which did not need complex instrument systems such as multi-tasking platforms. A perfect example of such an ingenious solution when performing an endoscopic myotomy is the principle of a peroral endoscopic myotomy (POEM) [32]. As opposed to using a complex multitasking endoscopic platform through the esophagus or the abdomen, a new and quite different access route was used, which required only a minimal amount of new endoscopic instruments [8,32].

The endoscopic resections of mucosal tumors or even tumors that have grown deeper into the gut wall are a challenging task, and the use of EMR has been attempted [1,2,33,34]. However, oncologic radicality requires a complete resection of the involved tissue and a correct histopathologic assessment afterward [1,2,3,33,34]. Endoscopic full-thickness resection (EFTR) is another solution to this task [1,2,3,33,34]. A sufficient grasping force, sufficient instrument tip maneuverability, some triangulation of the operating instruments, and necessary mobility of the scope are required to perform an EFTR [3,33,34]. Currently, this can be quite safely performed via the OTSC method [3,33,34]. However, the tumor size limits the endoscopic possibility depending on the used clip size. If EFTR is performed in a fashion in which the gut is opened during the resection, traction and countertraction, triangulation, and sufficient vision and overview during the procedure are necessary [10,11,12]. Consequently, in these cases, more specialized grasping tools such as those demonstrated here are very helpful [13,14,15,16,17,18,19,35,36,37,38,39,40,41,42,43,44,45,46,47,48,49,50,51].

Regarding more specialized functions, the Endo-dissector meets the need for the precise lifting of submucosal tissue for safe division during ESD in an ideal fashion [15]. The identification of the tissue strands to be divided, followed by the precise grasping and application of monopolar energy, allows for improved tissue handling compared to the alternative, i.e., the angulation of a needle knife, hook, and/or hybrid knife to sever the tissue, since with the latter energy instruments, the tissue strands may slide away during movement of the tip.

In addition, the specialized function of the OTSG is based on its design, with larger excavated branches and transparent material, making it able to pick up the debris, specimens, or foreign bodies under endoscopic vision and carry a certain volume of the debris and even some fluid for transport [18]. The latter two instruments respond to very special needs during endoscopic procedures, which may not be sufficiently fulfilled by most of the regular commercially available instruments.

The weak point of this analysis is the lack of objectivity of the assessment since we used our own team to evaluate instruments developed by the team. Indeed, the analysis is based on our single-center experience. Thus, this review may serve as a stimulus for other authors to more objectively compare these results. In addition, this presentation of the use of different grasping devices developed for different special purposes is intended to be more of a review of ideas, rather than a strict comparison.

Future innovations regarding instruments for advanced endoscopic procedures may involve robotic technology. The advantage of the robotic technique is the optimal maneuverability of instrument tips in a small space [52,53,54,55,56,57,58,59]. At first sight, this seems to fit excellently with the small intraluminal space in the gut, which may limit instrument action. However, endoscopists do use the advantage of the small space by using the narrow gut wall for guidance and to stabilize the position of the scope, which is also important. More clinical experience and technical developments are emerging with the robot systems used in flexible endoscopy [52,53,54,55,56,57,58,59].

## 5. Conclusions

In conclusion, with the emerging expansion of therapeutic endoscopy into the field of the endoscopic resections of gut lesions, the research and development of additional and specialized instruments with special features can solve the unmet needs in these sophisticated and advanced procedures. The latter can help to increase the indications for the need for endoscopic resections of gut lesions in the future. Major advances in the development of advanced endoscopic techniques are based on innovative instruments and new emerging concepts of procedural techniques.

## Figures and Tables

**Table 1:** Overview of the endoscopic instruments and grasping systems used for this analysis.

Instrument	Year of Publication
Endo-dissector	2013 [15]
Additional working channel (AWC) system	2019 [16]
External independent next-to-the-scope grasper	2023 [19]
Single-port overtube manipulator system (SPOT), 3D overtube system for ESD	2021 [17]
OTSG Xcavator™	2022 [18]

**Table 2:** Overview of the different experimental and clinical applications of the five chosen endoscopic instruments.

	Porcine Explant Model of Upper GI Tract	In Vivo Porcine Model	Human Clinical Experience after Initial Clinical Introduction and Publication
Endodissector (*)	10	5	8
AWC (**)	50	0	30
EINTS	32	10	0
SPOT	20	4	0
OTSG Xcavator (**)	10	0	>100

(*) Supporting company has stopped further activity. (**) Supporting company has launched the instrument on the market.

**Table 3:** Overview of the results of the analysis of different endoscopic grasping systems with a variety of special functions for advanced endoscopic therapies.

Critical Issues during ESD with Endoscopic Instruments	Standard Forceps	Endo-Dissector	AWC System (Using Standard Forceps)	EINTS Grasper	SPOT Work-Station	OTSG Xcavator
Traction/countertraction	+	+	++	+++	+++	+
Grasping force	+	++	+	+++	++	++
Independent instrument tip maneuverability	0	0	0	++	+++	0
Angulation/triangulation	0	0	+	++	+++	0
Mobility within GI tract	+++	+++	++	+	++1 arm +2 arms	++
Differentiated grasping abilities/special function	+	+++	++	+++	+++	+++

## Data Availability

Data are contained within the article.
